# Supplementary material for: Designing appropriate, acceptable and feasible community-engagement approaches to improve routine immunisation outcomes in low- and middle-income countries: A synthesis of 3ie-supported formative evaluations
Source: PLoS One. 2022 Oct 7;17(10):e0275278. doi: 10.1371/journal.pone.0275278 (PMC9543985; doi:10.1371/journal.pone.0275278)
Supplement: S1 Table — (DOCX) [file pone.0275278.s001.docx]

**S1 Table. Formative evaluation coding structure**

| Context | Barriers | |
| --- | --- | --- |
|  | Enablers | |
|  | Other ongoing programmes | |
| Existing evidence | Literature review | |
| Needs identification | Diagnostic and piloting work | |
| Intervention | Interaction with service providers | Capacity building and training |
|  |  | Supportive supervision |
|  |  | Mobilisation and recruitment |
|  |  | Incentives |
|  | Interaction with the community | Targeting |
|  |  | Participation |
|  |  | Supportive supervision for community health workers |
|  |  | Capacity building and training |
|  | Tools | Objective |
|  |  | Levers |
|  |  | Paper or technology-based tools |
|  | Research team roles and responsibility | Supportive supervision |
|  |  | Monitoring |
|  |  | Logistic support |
|  |  | Participant tracking |
|  | Intervention delivery | |
|  | Monitoring | |
| Study | Timeframe | |
|  | Methodology | |
|  | Site Selection | |
|  | Objective | |
|  | Sample | |
|  | Surveying | |
|  | Limitations | |
| Findings | Service provider | Acceptability |
|  |  | Take up |
|  |  | Feasibility |
|  |  | Cost effectiveness |
|  | Community | Acceptability |
|  |  | Take up |
|  |  | Feasibility |
|  |  | Equity |
|  |  | Gender |
|  |  | Knowledge |
|  | Health system | Barriers |
|  |  | Enablers |
| Stakeholder engagement and communication | Researcher or implementer specific factors | |
|  | Breadth and depth of engagement | |
|  | Budget | |
|  | Objectives | |
|  | Letter of Understanding | |
|  | Evidence uptake and use | |
| Implications | Programme | |
|  | Policy | |
|  | Research | |
