# Supplementary material for: Designing appropriate, acceptable and feasible community-engagement approaches to improve routine immunisation outcomes in low- and middle-income countries: A synthesis of 3ie-supported formative evaluations
Source: PLoS One. 2022 Oct 7;17(10):e0275278. doi: 10.1371/journal.pone.0275278 (PMC9543985; doi:10.1371/journal.pone.0275278)
Supplement: S2 Table — (DOCX) [file pone.0275278.s002.docx]

**S2 Table. Description of interventions**

| **Study** | **Intervention description** |
| --- | --- |
| Participatory action research (PAR) approach in Nigeria [5] | In Nigeria, researchers used the PAR approach to identify and address barriers to immunisation coverage and deliver the government’s Reaching Every Ward strategy in the Remo North local government area in Ogun State. The PAR approach worked with three groups of stakeholders, namely community members, health workers (both CHWs and FHWs) and local government officials, and created a tiered structure for collaboratively developing, implementing, and monitoring Joint Action Plans. |
| The Fifth Child Project in Ethiopia [6] | In Ethiopia, the International Rescue Committee (IRC), in collaboration with the London School of Hygiene and Tropical Medicine, mobilised frontline health workers (FHWs) and community health workers (CHWs) to conduct home visits and use a colour-coded calendar - Enat Mastawesha- as a job-aide for personalised counselling and improved communication with caregivers. The calendar also helped caregivers to keep track of upcoming perinatal appointments. To help with the effective tracking of defaulters, they also provided FHWs with a defaulter-tracing tool. Additionally, they engaged community (kebele) leaders in monitoring and kebele meetings attended by villagers and health workers to discuss issues with immunisation. |
| Collaborative community checklists for immunisation (CCCI) in Myanmar [7] | In Myanmar, the Burnet Institute, in collaboration with the Expanded Programme on Immunization (EPI), introduced service provider and caregiver checklists in three villages in Magway Region. The service provider checklist was an adaptation of the WHO immunisation session checklist [1]. The community checklist was the caregiver’s opportunity to provide feedback on health facility services, identify service delivery issues, and discuss these in a community forum where health providers participate. |
| Vaccine Indicator Reminder (VIR) bands in Nigeria and Pakistan [8,9] | In Nigeria’s Kebbi state and Pakistan’s Sindh province, researchers piloted the Vaccine Indicator Reminder (VIR) band-- a visual reminder for caregivers to vaccinate their children on time for DPT1-3. Encased in a silicone mould and worn as an anklet by the infant, the band comprised a time-strip indicator, which upon activation released a red dye calibrated to reach the endpoint when the next dose of vaccination became due. In Nigeria, CHWs and FHWs were given monetary incentives, whereas in Pakistan, only CHWs got them. The intervention also included extensive community engagement around the VIR bands. In Pakistan, the study recruited “champions” within the community to promote the bands. In Nigeria, traditional and religious leaders and CHWs were to promote the VIR bands in their communities. |
| Promoting Health Development Army among pastoralists in Ethiopia (Pastoralists Ethiopia) [10] | In Ethiopia, the Center for National Health Development explored the feasibility of adapting and extending the government’s community mobilisation platform, the Health Development Army (HDA), to pastoral communities. Health extension workers, Ethiopia’s FHWs, trained community women to become HDA members or CHWs. HDA members drive health-related behaviour change within their communities. Community women living in the same neighbourhood are organised into 1-5 networks, and four or five such networks federate to form a development team of 20-30 households in a sub-locality. The development teams are represented by a team leader from the community who works closely with FHWs and village leaders. |

**References**

1. World Health Organization. Immunization in practice: a practical guide for health staff – 2015 update. Geneva: World Health Organization; 2015.
