# Supplementary material for: Designing appropriate, acceptable and feasible community-engagement approaches to improve routine immunisation outcomes in low- and middle-income countries: A synthesis of 3ie-supported formative evaluations
Source: PLoS One. 2022 Oct 7;17(10):e0275278. doi: 10.1371/journal.pone.0275278 (PMC9543985; doi:10.1371/journal.pone.0275278)
Supplement: S3 Table — (DOCX) [file pone.0275278.s003.docx]

**S3 Table. Overview of formative evaluations included in the synthesis**

| Study | Primary questions | Methodology and data sources | | Stakeholders consulted | Type of engagement |
| --- | --- | --- | --- | --- | --- |
|  |  | **Quantitative** | **Qualitative** |  |  |
| CCCI Myanmar (Morgan et al. 2019) | - Intervention feasibility and acceptability amongst providers and communities. - Perceived effect of the intervention on knowledge and attitudes amongst caregivers and providers. - Perceived effect of the intervention on immunisation service quality and uptake amongst caregivers and providers. - Role of the intervention in identifying barriers to immunisation and engaging community members in addressing these. | Health facility data on immunisation, household survey | Key informant interviews, focused group discussions, in-depth interviews | Caregivers, pregnant women, local midwives, township medical officer, FHWs, community leaders, checklist assistants | ***Community engagement in intervention design:*** collaborating with the community and the healthcare providers on the design of the caregiver and provider checklists.  ***Engagement as the intervention:*** identifying community members to be volunteer checklist assistants and training them to provide support to caregivers in using the community checklists at immunisation sessions. |
| Pastoralists  Ethiopia (Teklehaimanot et al. 2019) | - Feasibility of implementing HDA or CHW network in pastoral communities. - Community acceptance of the intervention. - Intervention impact on immunisation rates. | Project monitoring data, household surveys | Focused group discussions, key informant interviews | Caregivers, health officials, FHWs, HDA or CHW network members and leaders, village health committee members, traditional leaders, kebele administration leaders | ***Community engagement in designing the intervention:*** soliciting community feedback on the design of the calendar.  ***Community engagement as the intervention:*** developing community buy-in through outreach.  ***Community engagement in implementation:*** involving community leaders in governance and decisions related to tracking and registering of immunisation defaulters. |
| Fifth Child Ethiopia (Demissie et al. 2019) | - Integration of intervention within the local health system. - Use of tools by FHWs in their interactions with caregivers.   Intervention performance in tracing immunisation defaulters. | Project monitoring data, health management information system data, household surveys | Semi-structured interviews, focused group discussions, observations | Caregivers, healthcare workers (FHWs and CHWs), community leaders and other members | ***Community engagement as the intervention:*** forming a new cadre of pastoral community health workers.  ***Community engagement in implementation:*** making HDA members and leaders responsible for running the weekly meetings and engaging all members in micro planning, implementation and monitoring of the programme. |
| PAR Nigeria (Akwataghibe et al. 2019) | - Effectiveness of the PAR approach in improving immunisation coverage and delivery of Reaching Every Ward strategy in the intervention areas.   Lessons from implementing the PAR approach and its potential in increasing immunisation access and utilisation in Nigeria. | Household survey, HMIS and health facility data | In-depth interviews, focused group discussions | Policy makers, local government officials, community leaders, health workers and caregivers | ***Community engagement as the intervention:*** developing Joint action plans as a downstream effect of mobilising the community for the PAR discussions.  ***Community engagement in implementation:*** involving the community in implementation and monitoring of the joint action plans. |
| VIR Pakistan (Rakhshani et al. 2019) | - Parental acceptance and compliance of the VIR band. - Accuracy of the VIR band/time-strip indicator.   Effectiveness of VIR bands as reminders to parents. | Project monitoring data, household survey | In-depth interviews, focused group discussions, social mapping | Caregivers, FHWs, CHWs, religious and political leaders and NGO representatives | ***Community engagement in design of the intervention:*** soliciting community feedback on the band design.  ***Community engagement as the intervention:*** developing community buy-in through engaging community volunteers and health workers. |
| VIR Nigeria (Obi-Jeff et al. 2019) | - Parental acceptance and compliance of the VIR band. - FHW ability to follow VIR band protocol to ensure it appropriate use. - Feasibility for health providers to incorporate VIR band in routine immunisation services. | Household survey at baseline, exit interviews at endline. | In-depth interviews, focused group discussions | State level policy makers, programme managers, healthcare workers, traditional birth attendants, caregivers, ward development committee members and other community members | ***Community engagement in design of the intervention:*** soliciting community feedback on the band design.  ***Community engagement as the intervention:*** developing community buy-in through engaging traditional and religious leaders, community volunteers and health workers. |
