# Supplementary material for: Designing appropriate, acceptable and feasible community-engagement approaches to improve routine immunisation outcomes in low- and middle-income countries: A synthesis of 3ie-supported formative evaluations
Source: PLoS One. 2022 Oct 7;17(10):e0275278. doi: 10.1371/journal.pone.0275278 (PMC9543985; doi:10.1371/journal.pone.0275278)
Supplement: Attachment — Submitted filename: renamed_ebdcd.pdf [file pone.0275278.s004.pdf]

**Reviewer's report: BM**

**Summary:** Thank you for the opportunity to peer review this important article. The article, '**Designing, appropriate, acceptable, and feasible community-engagement approaches to improve routine immunisation outcomes in low- and middle-income countries: a synthesis of 3ie-supported formative evaluations**.' Being a systematic review utilising inductive inquiry, this is in a way secondary data study. These results will contribute to the body of knowledge on correlates for demand of vaccination service, particularly in the area of community engagement.

**Overall impression:** The aim of the study was to synthesize qualitative findings from the six (6) 3ie-supported formative evaluations undertaken in Ethiopia, Myanmar, Nigeria, and Pakistan by International Initiative for Impact Evaluation (3ie) organisation in-order to:
- broaden the understanding of formative evaluations
- better understand community influencers

**Major issues:**
• **Positive note**: With the use of Critical Appraisal Skills Programme (CASP) checklist tool the authors followed the research methodology clearly and the documentation is well presented as a qualitative research with clear results, including documentation on study ethics. Formative evaluation structure is documented, and summary description of results has been illustrated in tables 2 and 3 of the supplementary material.

**Minor issues:**
Line 103: requires editing
Line 127: where is this figure 1 (was it uploaded separately)

**Recommendation:** • Accept article with minor changes/update/edits.
